# Lead-free LiNbO_3_ nanowire-based nanocomposite for piezoelectric power generation

**DOI:** 10.1186/1556-276X-9-4

**Published:** 2014-01-04

**Authors:** Byung Kil Yun, Yong Keun Park, Minbaek Lee, Nuri Lee, William Jo, Seongsu Lee, Jong Hoon Jung

**Affiliations:** 1 Department of Physics, Inha University, Incheon 402-751, Republic of South Korea; 2 Department of Physics, Ewha Womans University, Seoul 120-750, Republic of South Korea; 3 Neutron Science Division HANARO, Korea Atomic Energy Research Institute, Daejeon 305-353, Republic of South Korea

**Keywords:** High and stable electric power, Lead-free LiNbO_3_ nanowire, Nanocomposite nanogenerator

## Abstract

In a flexible nanocomposite-based nanogenerator, in which piezoelectric nanostructures are mixed with polymers, important parameters to increase the output power include using long nanowires with high piezoelectricity and decreasing the dielectric constant of the nanocomposite. Here, we report on piezoelectric power generation from a lead-free LiNbO_3_ nanowire-based nanocomposite. Through ion exchange of ultra-long Na_2_Nb_2_O_6_-H_2_O nanowires, we synthesized long (approximately 50 μm in length) single-crystalline LiNbO_3_ nanowires having a high piezoelectric coefficient (*d*_33_ approximately 25 pmV^-1^). By blending LiNbO_3_ nanowires with poly(dimethylsiloxane) (PDMS) polymer (volume ratio 1:100), we fabricated a flexible nanocomposite nanogenerator having a low dielectric constant (approximately 2.7). The nanogenerator generated stable electric power, even under excessive strain conditions (approximately 10^5^ cycles). The different piezoelectric coefficients of *d*_33_ and *d*_31_ for LiNbO_3_ may have resulted in generated voltage and current for the *e*_33_ geometry that were 20 and 100 times larger than those for the *e*_31_ geometry, respectively. This study suggests the importance of the blending ratio and strain geometry for higher output-power generation in a piezoelectric nanocomposite-based nanogenerator.

**PACS:**

77.65.-j; 77.84.-s; 73.21.Hb

## Background

Lead-based piezoelectric materials, such as Pb(Zr,Ti)O_3_ and Pb(Mg,Nb)O_3_-PbTiO_3_, have been utilized for the last several decades in actuators, transducers, and sensor applications [1]. As the restriction of hazardous substances becomes an emerging issue, however, much attention has been paid to lead-free piezoelectric materials having a perovskite structure [2]. Among the candidates to replace toxic lead-based piezoelectric materials, alkaline niobates, such as (K,Na,Li)NbO_3_, are regarded as one of the most appropriate materials due to their high Curie temperature, piezoelectric coefficient, and electromechanical coupling coefficient [3,4].

In addition to nanoelectromechanical system (NEMS) applications, one of the most challenging applications of nanosize lead-free piezoelectric materials is the nanogenerator, which can effectively convert ubiquitous mechanical vibrations into electricity [5]. Due to the low power consumption of modern devices, lead-free piezoelectric nanostructure-based nanogenerators could be a powerful alternative to batteries. Until recently, several nanogenerators have been reported using BaTiO_3_, ZnSnO_3_, Pb(Zr,Ti)O_3_, Pb(Mg,Nb)O_3_-PbTiO_3_, and (K,Na)NbO_3_[6-11]. In particular, piezoelectric nanocomposite devices, in which piezoelectric nanostructures are mixed with flexible polymers, have exhibited relatively easy, cost-effective fabrication, and high-power generation [9-13]. In a flexible nanocomposite-based nanogenerator, important parameters to increase the output power include using long nanowires with high piezoelectricity and decreasing the dielectric constant of the nanocomposite [9].

In this paper, we report on piezoelectric power generation from a lead-free LiNbO_3_ nanowire-based composite device. As for the nanogenerator applications, LiNbO_3_ has several merits such as small dielectric constant, relatively high piezoelectric constant, and thermal stability [14,15]. Through successful ion exchange in micro-porous Na_2_Nb_2_O_6_-H_2_O nanowires, we synthesized long (approximately 50 μm) LiNbO_3_ nanowires having high piezoelectricity (approximately 25 pmV^-1^). By mixing LiNbO_3_ and poly(dimethylsiloxane) (PDMS) (in a volume ratio of 1:100, respectively), we fabricated a flexible nanogenerator having a low dielectric constant for the *e*_33_ and *e*_31_ geometries. For a similar value of strain, we note that the open-circuit voltage and closed-circuit current for the *e*_33_ geometry were 20 and 100 times larger than those for the *e*_31_ geometry, respectively. For up to 10^5^ cycles of strain, we observed that the generated power was quite stable; the dielectric constant and electric loss did not change significantly.

## Methods

High-quality LiNbO_3_ nanowires were synthesized using a three-step procedure. First, we obtained microporous Na_2_Nb_2_O_6_-H_2_O nanowires by a hydrothermal method. NaOH (12 M) was dissolved in 20 mL of distilled water; 0.113 M of Nb_2_O_5_ was then added to the NaOH solution. The solution was stirred and transferred into a 25-mL Teflon lining in a stainless steel autoclave to undergo a hydrothermal reaction at 120°C for 5 h. In the second step, we obtained Li_2_Nb_2_O_6_-H_2_O nanowires using the ion-exchange method. LiCl (20 M) was dissolved in 20 mL of distilled water. Na_2_Nb_2_O_6_-H_2_O nanowires were added to the LiCl solution. After stirring for 20 h, the stirred solution was filtered, washed with distilled water, and dried at 80°C for 12 h. In the third step, LiNbO_3_ nanowires were obtained after heating the ion-exchanged Li_2_Nb_2_O_6_-H_2_O nanowires at 500°C for 2 h.

The crystalline structure of the nanowires was characterized by high-resolution X-ray diffraction (HR-XRD), field-emission scanning electron microscopy (FE-SEM), and field-emission transmission electron microscopy (FE-TEM) measurements. To characterize the detailed crystal structure and symmetry, we performed neutron diffraction measurements and a Rietveld analysis. We used piezoresponse force microscopy (PFM) to investigate the piezoelectricity and piezoelectric/ferroelectric domains of the LiNbO_3_ nanowires. The PFM measurements were performed using an atomic force microscope at 1 V and 73 kHz. To scan the surface, we used Pt/Ir-coated tips and a force constant of 3 Nm^-1^. Before scanning, we thoroughly dispersed and tightly attached the nanowires to the Pt-coated Si substrate using a polymer (5 wt.% poly(vinylpyrrolidone) dissolved in ethanol). The LiNbO_3_ nanowires were then coated with 10-nm-thick Pt to obtain a uniform electric field and to minimize electrostatic effects.

To fabricate the nanocomposite nanogenerator, the LiNbO_3_ nanowires were thoroughly mixed with PDMS at a volume ratio of 1:100. (We noted that LiNbO_3_ nanowires were not mixed well with PDMS for an increased volume ratio of 2:100.) Small amounts of the mixture were spin-coated onto an Au/Cr-coated Kapton polyimide film at 500 rpm for 10 s. The 25-nm-thick Au and 10-nm-thick Cr films were deposited onto the Kapton film by thermal evaporation. Another Au/Cr-coated Kapton film was attached to the top surface of the spin-coated LiNbO_3_-PDMS composite for the electrode. Finally, polyester (PS) film was attached to the bottom Kapton film. The thicknesses of the Kapton and PS films were 125 and 500 μm, respectively. We applied an electric field of approximately 100 kV · cm^-1^ for electric poling at room temperature [16].

To measure the Young’s modulus of the LiNbO_3_-PDMS composite, we used a nanoindenter with a Berkovich tip, and applied the continuous stiffness measurement option. A linear motor was used to periodically apply and release compressive force at a frequency of 0.8 Hz. The pushing and bending amplitudes were varied over the course of the measurement. The output signal of the piezoelectric device was recorded by low-noise voltage and current preamplifiers.

## Results and discussion

**Figure 1:**
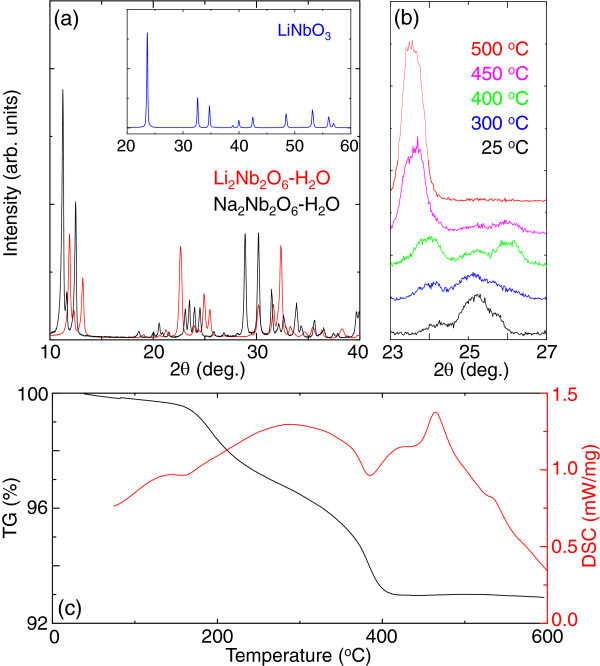
**Phase transformation from Li**_**2**_**Nb**_**2**_**O**_**6**_**-H**_**2**_**O to LiNbO**_**3**_**.** High-resolution X-ray diffraction (HR-XRD) patterns of Li_2_Nb_2_O_6_-H_2_O at **(a)** room temperature and **(b)** elevated temperatures. In (a), we show the XRD patterns of Na_2_Nb_2_O_6_-H_2_O and LiNbO_3_ for comparison. **(c)** Thermogravimetric (TG) and differential scanning calorimetry (DSC) results for Li_2_Nb_2_O_6_-H_2_O.

Microporous Na_2_Nb_2_O_6_-H_2_O nanowires seem to be an excellent template for ion exchange [17]. Due to the smaller ionic size of the lithium ion (Li^+^) compared with the sodium ion (Na^+^), as well as the excessive amount of LiCl (i.e., approximately 20 M), all of the Na^+^ appeared to be involved in the exchange with Li^+^ in Na_2_Nb_2_O_6_-H_2_O. Figure 1a compares the XRD pattern of Li_2_Nb_2_O_6_-H_2_O and Na_2_Nb_2_O_6_-H_2_O. The overall XRD pattern of Li_2_Nb_2_O_6_-H_2_O was quite different from that of Na_2_Nb_2_O_6_-H_2_O. From an inductive-coupled plasma (ICP) measurement of Li_2_Nb_2_O_6_-H_2_O, we did not find any trace of Na^+^ within the experimental limits. These results imply that crystalline Li_2_Nb_2_O_6_-H_2_O could be obtained from Na_2_Nb_2_O_6_-H_2_O through an ion exchange process.

In Figure 1b, we show *in-situ* XRD patterns of Li_2_Nb_2_O_6_-H_2_O at elevated temperatures. The diffraction patterns of Li_2_Nb_2_O_6_-H_2_O were significantly modified with an increase in temperature, especially above 400°C, and exhibited an irreversible phase transformation. In the inset of Figure 1a, we show the XRD pattern after heat treatment of Li_2_Nb_2_O_6_-H_2_O. We note that the XRD pattern obtained after heat treatment was well indexed by LiNbO_3_. To the best of our knowledge, this is the first report for the synthesis of LiNbO_3_ nanowire through ion exchange and subsequent heat treatment.

To gain insight into the phase transformation from Li_2_Nb_2_O_6_-H_2_O to LiNbO_3_, we show the thermogravimetric (TG) and differential scanning calorimetry (DSC) results in Figure 1c. The mass of Li_2_Nb_2_O_6_-H_2_O changed significantly near 400°C and was accompanied by endothermic reactions at the same temperature. After the endothermic reactions, an exothermic reaction occurred near 460°C without a noticeable change in the mass. Comparing the well-known phase transformation mechanism from Na_2_Nb_2_O_6_-H_2_O to NaNbO_3_[18], the peaks at 400°C and 460°C corresponded well to the dehydration of H_2_O from Li_2_Nb_2_O_6_-H_2_O and the structural transformation from Li_2_Nb_2_O_6_ to LiNbO_3_, respectively. (The broad change in the mass near 220°C seems to have originated from the desorption of surface/lattice-absorbed hydroxyl defects [19]).

**Figure 2:**
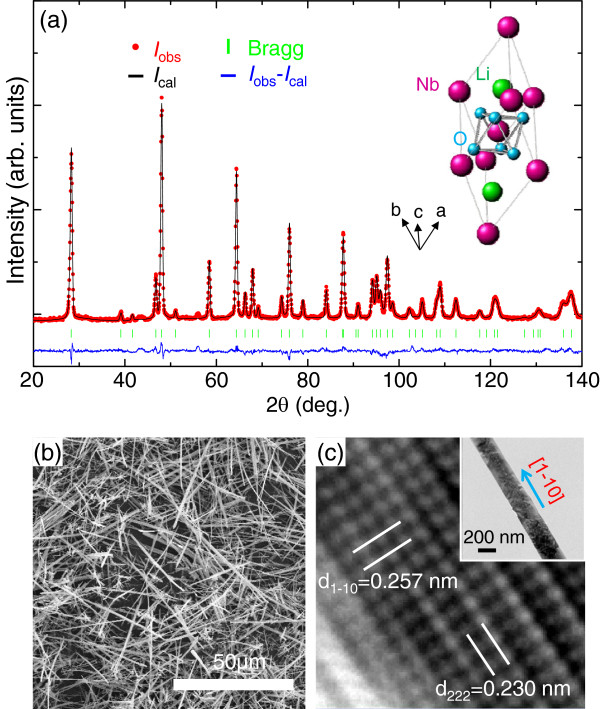
**Structural characterization of LiNbO**_**3**_**. (a)** Rietveld analysis of neutron diffraction patterns of LiNbO_3_. The red dots represent the observed intensity. The black lines represent the calculated intensity. The blue line corresponds to the difference between the observed and calculated intensities. The green line shows the Bragg reflection. In the inset of **(a)**, we show the crystal structure of LiNbO_3_. **(b)** Field-emission scanning electron microscopy (FE-SEM) and **(c)** high-resolution transmission electron microscopy (HR-TEM) images of LiNbO_3_. In the inset of **(c)**, we show a medium-resolution TEM image of a LiNbO_3_ nanowire.

Due to the light Li ions, we used neutrons rather than X-rays to determine the detailed crystal structure of LiNbO_3_. Figure 2a shows a Rietveld analysis of the neutron diffraction pattern of LiNbO_3_. The neutron diffraction pattern of LiNbO_3_ was well-fit by the trigonal structure (*a* = 5.488 Å, *α* = 55.89°) with *R3c* symmetry. The resulting lattice constant (angle) of the LiNbO_3_ nanostructure was slightly smaller (larger) than that of the LiNbO_3_ single crystal (*a* = 5.492 Å, *α* = 55.53°) [20]. Based on the Rietveld analysis, we show the crystal structure of LiNbO_3_ in the inset of Figure 2a. The Nb ions in the NbO_6_ octahedra shifted toward the [111] direction, hence initiating the spontaneous formation of electric polarization without applying an electric field.

Figure 2b,c shows FE-SEM and HR-TEM images of LiNbO_3_, respectively. All of the LiNbO_3_ samples had nanowire morphology, with a high aspect ratio of 160 to 600 (width 100 to 250 nm; length 40 to 60 μm). Note that the LiNbO_3_ nanowires, synthesized using the molten salt method, had a relatively short length (<10 μm) [21]. The clear lattice fringe indicated the single-crystalline quality of the LiNbO_3_ nanowires. Based on the Rietveld analysis, the LiNbO_3_ nanowires appeared to grow along the [1-10] direction.

**Figure 3:**
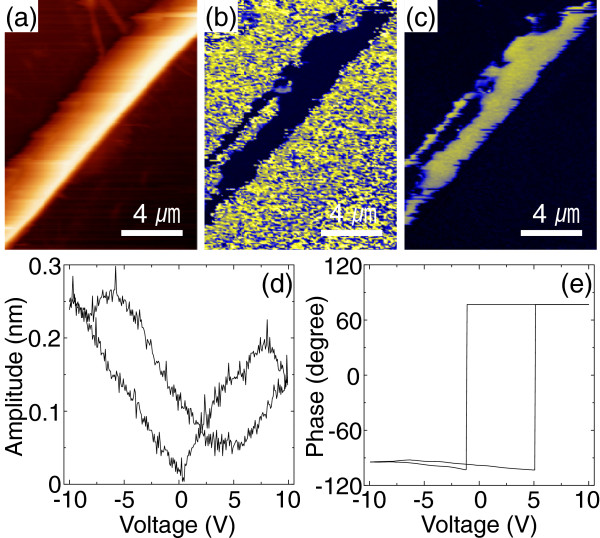
**Piezoelectricity/ferroelectricity of the LiNbO**_**3 **_**nanowire. (a)** Topography, **(b)** piezoelectric amplitude, and **(c)** piezoelectric phase for a LiNbO_3_ nanowire. Applied voltage dependences of **(d)** piezoelectric amplitude and **(e)** piezoelectric phase.

To investigate the piezoelectricity of the LiNbO_3_ nanowires, we used PFM. Figure 3a,b,c shows the topography, amplitude, and phase of the piezoelectric response of a single LiNbO_3_ nanowire, respectively. The brightness of the amplitude map represents the strength of the piezoelectric response; the contrast of the phase map corresponds to the direction of the electric polarization in the nanowire. From Figure 3b,c, the piezoelectric domains in the LiNbO_3_ nanowire were clearly evident.

Figure 3d,e shows the switching of the piezoelectric/ferroelectric amplitude and phase with the application of direct-current (dc) voltage. An abrupt change in the phase suggests the switching of domains in LiNbO_3_, which is generally associated with ferroelectric behavior [22]. We estimated the piezoelectric coefficient *d*_33_ value from the linear portion of the piezoresponse amplitude signal as approximately 25 pmV^-1^.

**Figure 4:**
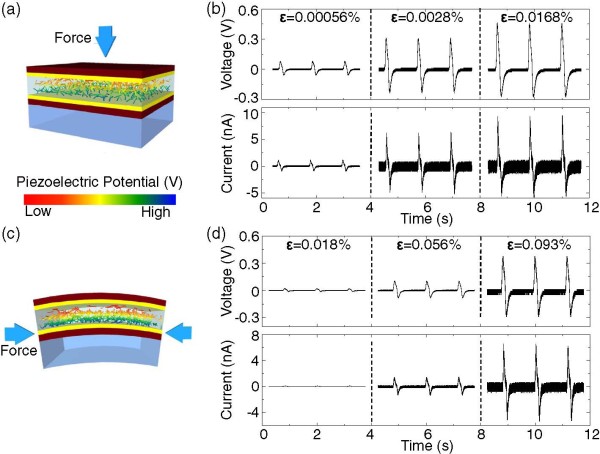
**Schematic diagram and power generation for the LiNbO**_**3**_**-PDMS composite nanogenerator.** Schematic diagram of the LiNbO_3_-PDMS composite nanogenerator for **(a)***e*_33_ and **(c)***e*_31_ geometries. Dark brown, yellow, and light blue represent the Kapton film, Au/Cr electrode, and PS film, respectively. The rainbow color of the LiNbO_3_ nanowires represents the piezoelectric potential after the stress application. The open-circuit voltage (*V*) and closed-circuit current (*I*) at selected strains for **(b)***e*_33_ and **(d)***e*_31_ geometries.

After confirming the piezoelectricity/ferroelectricity of the LiNbO_3_ nanowire, we fabricated a composite nanogenerator for the *e*_33_ and *e*_31_ geometries, as schematically shown in Figure 4a,c, respectively. Even though the LiNbO_3_ nanowires were randomly distributed inside the PDMS polymer, the piezoelectric/ferroelectric domains could be vertically aligned after applying a strong electric field for poling. If we were to apply stress, then the nanowires would be subjected to compressive strain, which would induce a piezoelectric potential due to the piezoelectricity of LiNbO_3_. To screen the piezoelectric potential, positive and negative charges would accumulate at the top and bottom electrodes, respectively. Once the strain is released, the piezoelectric potential should diminish and the accumulated charges should move back in the opposite direction. Therefore, the continuous application and release of the strain will result in an alternating voltage and current [23].

To quantify the strain (*ϵ*), we used Young’s modulus, *Y*, of the LiNbO_3_-PDMS, Kapton, and PS films, having values of 0.87, 2.5, and 3.25 GPa, respectively [24]. The strain for the *e*_33_ geometry was then calculated using the equation *ϵ* = *P*/*Y*, where *P* represents the applied pressure. To quantify the strain for the *e*_31_ geometry, we calculated the strain neutral line from the equation Σ*Y*_*i*_*t*_*i*_*y*_*i*_ = 0 (for *i* = 1 to 4), where *t* and *y* represent the thickness of each layer and the distance from the strain neutral line to the center of each layer, respectively. The strain for the *e*_31_ geometry was obtained using the equation ϵ = 2 *t*′ × *h*/(*a*^2^ + *h*^2^), where *a*, *h*, and *t*′ represent the half-width of the arc, the height of the arc, and the distance from the strain neutral line to the center of the LiNbO_3_-PDMS composite layer, respectively [25].

Figure 4b,d shows the open-circuit voltage and closed-circuit current obtained for the *e*_33_ and *e*_31_ geometries, respectively. Through the polarity reversal test, we confirmed that the signals originated from the piezoelectricity of LiNbO_3_. With an increase in the strain, both the voltage and current increased as well. We note that the obtained voltage (current) for the *e*_33_ geometry was almost 20 times (100 times) larger than that for the *e*_31_ geometry for a similar value of the strain. For example, the open-circuit voltage and closed-circuit current (current density) for *e*_33_ with *ϵ* = 0.0168% were 0.46 V and 9.11 nA (4.64 nA · cm^-2^), respectively; whereas, for *e*_31_ with *ϵ* = 0.018%, values of 0.02 V and 0.09 nA (0.044 nA · cm^-2^) were obtained, respectively. Note that due to the low output voltage and current for *e*_31_, we could not detect a signal for strain lower than *ϵ* = 0.018%. The electric power generated from the piezoelectric nanostructures was affected by the piezoelectric coefficient, dielectric constant, and strained length of the nanowire [9]. All of the other parameters were the same for both the *e*_33_ and *e*_31_ geometries, which implied that the significant difference in power generation was related to the different piezoelectric coefficients of *d*_33_ = 27 pmV^-1^ and *d*_31_ = 4.3 pmV^-1^ for LiNbO_3_[26].

**Figure 5:**
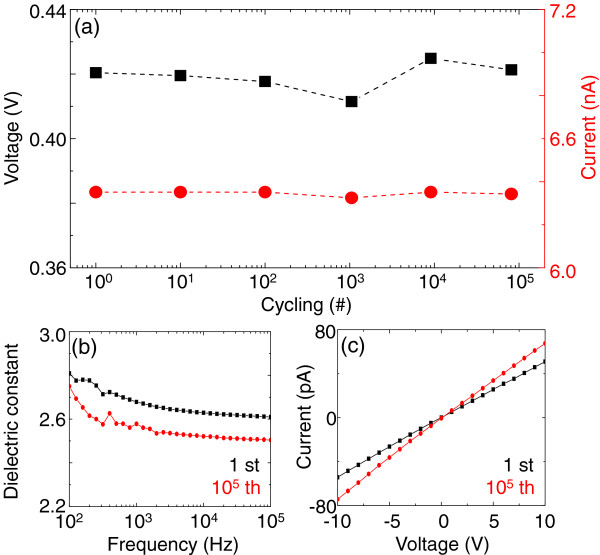
**Stability of the LiNbO**_**3**_**-PDMS composite nanogenerator. (a)** Cycling number-dependent open-circuit voltage and closed-circuit current of the LiNbO_3_-PDMS composite nanogenerator. **(b)** Dielectric constant and **(c)** current–voltage (*I-V*) characteristics before and after 10^5^ cycles of excessive strain.

The LiNbO_3_-PDMS-based composite nanogenerator for the *e*_33_ geometry generates stable power even for excessive strain. In Figure 5a, we show the push-pull cycling number dependence of the open-circuit voltage and closed-circuit current. Over a period of 22 h, we continuously applied a compressive strain of up to 10^5^ cycles. Within ±1%, the open-circuit voltage and closed-circuit current were quite stable. The stability of the dielectric constant and electric loss are shown in Figure 5b,c, respectively. The dielectric constant and current–voltage (*I-V*) characteristics were similar before and after the application of excessive strain (approximately 10^5^ cycles).

In the LiNbO_3_-PDMS composite nanogenerator, stable power generation depended on the mixing ratio. LiNbO_3_ has high piezoelectricity, but is fragile and lossy. In contrast, PDMS has flexibility and a low dielectric constant, but no piezoelectricity. Nearly the same power generation, dielectric constant, and loss after excessive strain suggest that our LiNbO_3_-PDMS composite nanogenerator was quite stable; this was attributed to the low volume ratio of LiNbO_3_ inside the PDMS (approximately 1%). If the volume ratio of LiNbO_3_ were to increase, then the power generation would increase as well at the expense of a larger dielectric constant; however, the composite devices may become fragile and lossy. Therefore, we suggest that optimization of the mixing ratio is crucial for the application of a lead-free piezoelectric composite nanogenerator.

## Conclusions

We report a lead-free LiNbO_3_ nanowire-based nanocomposite for piezoelectric power generation. Through the ion exchange of Na_2_Nb_2_O_6_-H_2_O, we synthesized long (approximately 50 μm) single-crystalline LiNbO_3_ nanowires having a high piezoelectric coefficient (approximately 25 pmV^-1^). By blending LiNbO_3_ and PDMS polymer at a volume ratio of 1:100, we fabricated a flexible nanocomposite nanogenerator. For a similar strain, the piezoelectric power generation for the *e*_33_ geometry was significantly larger than that for the *e*_31_ geometry due to the difference in the *d*_33_ and *d*_31_ piezoelectric coefficients of LiNbO_3_. For up to 10^5^ cycles of excessive strain, we observed that the output power, dielectric constant, and loss were quite stable. Optimization of the mixing ratio between lead-free piezoelectric materials and flexible polymers is an important factor to consider in the application of an energy-harvesting nanogenerator.

## Competing interests

The authors declare that they have no competing interests.

## Authors’ contributions

BKY and YKP prepared the nanowire and performed the XRD, TG, DSC, SEM, and TEM measurements. BKY and ML fabricated the nanocomposite nanogenerator and tested the performance. NL and WJ carried out the PFM measurements and analysis. BKY and SL performed neutron diffraction measurements and the Rietveld analysis. JHJ designed the work and wrote the manuscript. All authors read and approved the final manuscript.
